# Hugh Hadfield Carleton

**Published:** 1962-01

**Authors:** 


### HUGH HADFIELD CARLETON

Dr. H. H. Carleton, past President of the Bristol Medico-Chirugical Society and Consulting Physician to the United Bristol Hospitals, died on 17th July at the age of 80. Hugh Carleton was born at Warrington and was the son of the Rev. E. C. E. Carleton. After taking a first in Natural Sciences at Keble College, Oxford, he was a clinical student at St. Thomas's Hospital and graduated B.M., B.Ch. in 1907. He proceeded D.M. in 1914, was admitted M.R.C.P. in 1919, and elected F.R.C.P. in 1935. He contracted pulmonary tuberculosis early in his career, which aroused his interest in this disease and in chest diseases in general. For a time he was medical superintendent of the Queen Alexandra Sanatorium, Davos Platz. While there he published one of his first papers on some technical aspects of artificial pneumothrax. He served with the R.A.M.C. during the 1914–18 war, and it was probably his own experience during this time, combined with Arthur Hurst's work on shell shock that aroused his interest in the psycho-neuroses which was to persist and develop for the rest of his life. At the end of the war he settled in Bristol and was elected a member of the Bristol Medico-Chirurgical Society in 1921. From then until his presidency in 1945–46 he played an important and valuable role in the affairs of the Society. The communications which he gave at meetings of the Society illustrate his main clinical interests—radiology in pulmonary diseases, the problem of haemoptysis, hysteria, speech disorders, disorders of movement, and trigeminal neuralgia. His early experience with the technique of artificial pneumothorax combined with natural manual dexterity made him an accomplished adept at injection of the Gasserian ganglion. Perhaps his most important publication was a joint paper with R. G. Gordon in Brain in 1923 on hysterical pain.

He was elected assistant physician to the Bristol General Hospital in 1923, became a full physician in 1933, and on the amalgamation of the Royal Infirmary and General Hospital in 1940, physician to the Bristol Royal Hospital. He served both hospitals and the medical school with loyalty and devotion until his retirement in 1946. For several years after this he kept in touch with clinical neurology by means of weekly visits to the neurosurgical unit at Frenchay. These visits he much enjoyed and they were also greatly appreciated by the members of the unit. Hugh Carleton was a sound physician, who always took a real interest in his patients. Keenly interested in teaching, he was held in high esteem and affection by his students who much enjoyed his ward rounds, enlivened as they were by a rather dry and subtle form of humour. His greatest contribution to the Bristol Medical School was undoubtedly the interest he aroused and the new concept which he brought to bear on the problems of the neuroses and what is now called psychosomatic medicine. The hospital and medical School owe him a very considerable debt.

He had several hobbies including carpentry and work in his small orchard at Nailsea, but his abiding interest was an amateur but very knowledgeable study of philosophy. Happily he was able to enjoy reading almost to the end, and this was a great source of comfort in his later years, especially after the death of his wife. His interest in philosophy led to the choice of subject of his Presidential address to the Society in 1945 on Nature and Science. In this he made a careful and critical study of scientific materialism and a plea to scientists to be moderate and tolerant. Hugh Carleton was an extremely kind and generous colleague, as one who was for some years his assistant physician knows only too well. We shall remember him as a good physician, a valued member of the Society, and a much loved companion.

